# Bleeding risk assessment for venous thromboembolism prophylaxis

**DOI:** 10.1590/1677-5449.200109

**Published:** 2021-04-28

**Authors:** Maria Chiara Chindamo, Marcos Arêas Marques

**Affiliations:** 1 Universidade Federal do Rio de Janeiro – UFRJ, Rio de Janeiro, RJ, Brasil.; 2 Hospital Barra D'Or - Rede D'Or São Luiz , Rio de Janeiro, RJ, Brasil.; 3 Universidade do Estado do Rio de Janeiro – UERJ, Rio de Janeiro, RJ, Brasil.

**Keywords:** venous thrombosis, pulmonary embolism, prophylaxis, hemorrhage, patient safety, risk assessment

## Abstract

Venous thromboembolism (VTE) is one of the main preventable causes of morbidity and mortality in hospitalized patients and fatal pulmonary embolism (PE) may be its first manifestation. Several national and international guidelines recommend using risk assessment models for prescription of VTE prophylaxis in hospitalized patients. Despite evidence and guidelines supporting VTE prevention, use of VTE prophylaxis in hospitalized patients remains suboptimal, which may be because of low awareness of the benefits of VTE prophylaxis, but might also reflect fear of bleeding complications in these patients, since this constitutes one of the main reasons for underutilization of thromboprophylaxis worldwide. Bleeding risk assessment is therefore necessary for adequate prophylaxis prescription and should be carried out concurrently with assessment of the risk of thrombosis. The purpose of this review is to highlight the importance of jointly assessing risk of VTE and risk of bleeding in hospitalized patients.

## INTRODUCTION 

Venous thromboembolism (VTE) is a major preventable cause of morbidity and mortality in hospitalized patients.^1^^,^ 2 The first manifestation of VTE is often fatal pulmonary embolism (PE), which can be responsible for up to 10% of all in-hospital mortality.^2^^,^ 3 Hospitalized patients can be at risk of VTE because of acquired or hereditary factors, such as obesity, cancer, previous VTE, thrombophilias, trauma, surgery, acute myocardial infarction, stroke, advanced age, congestive heart failure, acute infection, immobility, and admission to intensive care, among other factors.^4^^-^ 6

National and international guidelines recommend use of risk assessment models (RAM) for selection of pharmacological or mechanical prophylaxis in clinical,^7^^-^ 14 surgical,^8^^,^ 15 ^,^ 16 or obstetric patients,^10^ targeting better prevention strategies. However, VTE risk cannot be assessed in isolation. The risk of bleeding must also be assessed concurrently when the appropriate thromboprophylaxis strategy is being evaluated, since it can be induced or exacerbated by anticoagulants.^6^^,^ 7 ^,^ 9

Even though many studies have reported low rates of bleeding related to pharmacological prophylaxis,^11^^,^ 17 ^,^ 18 fear of hemorrhagic events is one of the main reasons for its underutilization worldwide.^6^ Identification of conditions involving a potential risk of bleeding and implementation of RAM are therefore essential to ensure correct use of thromboprophylaxis.^19^

The objective of this review is to highlight the importance of concurrent assessment of VTE risk and bleeding risk in hospitalized patients.

## ASSESSMENT OF THROMBOSIS RISK *VS.* BLEEDING RISK

There are many different VTE RAMs available, both for clinical and surgical patients, providing guidance on the principal thromboprophylaxis recommendations, based on risk stratification.^7^^-^ 16 The best assessment model has not yet been defined.^19^ When conducting VTE risk stratification, a model should be used that has been validated for the population in question and should be applied systematically at the key stages of care: hospital admission, transfer between sectors, and hospital discharge. This last assessment is particularly important in patients who still have risk factors for VTE at discharge, such as, for example, prolonged immobility.^2^ Choice of the best thromboprophylaxis strategy should simultaneously consider risk of VTE and the potential risk of bleeding.^20^^,^ 21

**Figure 1:**
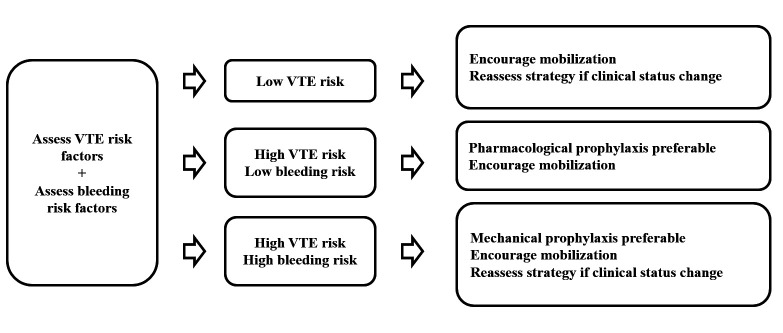
Recommendations for venous thromboembolism (VTE) prophylaxis by VTE risk *vs.* bleeding risk stratification. Adapted from: National Institute for Health and Care Excellence – NICE. NG89.^21^

The following are considered absolute contraindications to anticoagulants: severe or potentially fatal active bleeding, or active bleeding that is irreversible with medical or surgical intervention, including any active bleeding at critical sites (intracranial, pericardiac, retroperitoneal, intraocular, intraarticular, and intraspinal), malignant uncontrolled arterial hypertension, uncompensated severe coagulopathy, platelet dysfunction or severe primary hemostasis disorders, persistent thrombocytopenia (< 20,000/mm^3^), and high-risk invasive procedures in critical areas, such as lumbar puncture and spinal anesthesia in patients whose surgical procedures are scheduled for the next 6 to 12 hours.^20^ Other factors associated with increased risk of bleeding include heparin-induced thrombocytopenia (HIT), concomitant use of platelet antiaggregants and/or nonsteroidal anti-inflammatories, and renal dysfunction, particularly when anticoagulants subject to renal clearance are used (low molecular weight heparin [LMWH] and fondaparinux).^15^ For patients with creatinine clearance < 30 mL/min, it is recommended that the LMWH dose be reduced, anticoagulant activity be monitored, or unfractionated heparin (UFH) be used as a substitute.^4^ Regular reviews of both risks, especially when there are changes in clinical status, facilitate choice of the best prophylaxis strategy^21^ (Figure 1).

### VTE risk assessment models

The main RAMs for VTE in clinical patients include the Brazilian VTE Prevention Guidelines for hospitalized clinical patients^9^ and the Padua,^11^ Geneva,^13^ and IMPROVE (International Medical Prevention Registry on Venous Thromboembolism)^12^ scores. The Caprini^15^ and Rogers^16^ scores are recommended for assessment of surgical patients, defining VTE risk on the basis of patient characteristics and the profile of each type of surgery. Women admitted to hospital during pregnancy, puerperium, or during the 6 weeks after a miscarriage or aborted pregnancy should be assessed for pharmacological prophylaxis.^21^ The RAM most widely used for this patient profile was developed by the Royal College of Obstetricians and Gynecologists (RCOG).^10^^,^ 21

Although it is recommended that risk of VTE *vs.* risk of bleeding should be assessed concurrently as part of care for hospitalized patients, there are few RAMs for bleeding in the context of VTE prophylaxis.^19^ Few RAMs combine these two characteristics^9^^,^ 12 ^,^ 21 (Table 1).

**Table 1:** List of venous thromboembolism (VTE) and bleeding risk assessment models (RAM) according to study population.^9^^-^ 16 ^,^ 21

**RAM**	**Types of patient**	**Risk of bleeding**	**Prophylaxis Recommendation**
Caprini, 2005	Surgical	-	X
Geneva, 2006	Medical	-	X
Rogers, 2007	Surgical	-	X
Brazilian guidelines, 2007	Medical	X	X
Padua, 2010	Medical	-	X
IMPROVE, 2011	Medical	X	X
UK RCOG, 2015	Obstetric	-	X
NICE NG89, 2018	Medical /surgical	X	X

This situation is very different to what is found in relation to bleeding risk assessment in the context of full anticoagulation for prevention of the thromboembolic phenomena of atrial fibrillation or for treatment of VTE. Ten bleeding RAMs with this objective are available. Six of them are applicable to patients using oral anticoagulants for atrial fibrillation (ABC,^22^ ORBIT,^23^ ATRIA,^24^ HAS-BLED,^25^ HEMORR_2_HAGES,^26^ and Shireman^27^), three for anticoagulant VTE treatment (VTE-BLED,^28^ Ruiz-Gimenez,^29^ and Kuijer^30^), and one represents a mixed model (OBRI^31^). These scores identify situations of increased bleeding risk associated with full anticoagulation and support implementation of strategies that help to minimize the risk of hemorrhage by intervening in modifiable risk factors.^32^

## RISK OF BLEEDING WITH PHARMACOLOGICAL PROPHYLAXIS

### Assessment of bleeding risk in medical patients

#### IMPROVE *Bleeding Risk Score*

The principal RAM for bleeding associated with pharmacological prophylaxis in hospitalized medical patients is the IMPROVE Bleeding Risk Score.^6^^,^ 7 Decousus et al.^6^ used multivariate analysis to identify and score factors at hospital admission that were associated with risk of bleeding in acutely ill medical patients. Based on the IMPROVE data,^12^ these authors conducted an observational multicenter study developed to assess VTE prophylaxis standards in more than 15,000 medical patients, determined the incidence of bleeding, and identified factors at admission that were associated with risk of bleeding.^6^ Major bleeding was defined as fatal bleeding and/or symptomatic bleeding in a critical area or organ, and/or bleeding causing a ≥ 2 g/dL fall in hemoglobin or leading to transfusion of two or more units of packed red blood cells.^6^ Bleeding was defined as not major but still clinically relevant if there was gastrointestinal hemorrhage, macroscopic hematuria with duration > 24 h, substantial epistaxis requiring intervention, epistaxis that was recurrent and/or with duration of at least 5 minutes, extensive hematoma (> 5 cm in diameter), intraarticular bleeding, menorrhagia or metrorrhagia, or other types of important bleeding requiring medical intervention.^6^^,^ 33

The cumulative incidence of hospital bleeding, defined as major and non-major bleeding up to 14 days after the admission, was 3.2% (1.2% major bleeding and 2.0% non-major, but clinically relevant bleeding).^6^

Risk factors at admission that were independently associated with risk of bleeding were:^6^ active gastroduodenal ulcer, bleeding during the 3 months preceding admission, and platelet count < 50,000/mm.^3^ Other risk factors for bleeding included advanced age, liver and/or kidney failure, admission to an intensive care unit, presence of a central venous catheter, rheumatic disease, cancer, and male sex, which were also factors related to increased risk of VTE.^6^ Each of the factors above were included in the RAM with appropriate weighting (Table 2). The authors also developed an online resource that can be used to assess risk of bleeding.^34^

**Table 2:** IMPROVE Bleeding Risk Score.

**Risk factors**	**Score**
Active gastroduodenal ulcer	4.5
Hemorrhage 3 months before admission	4
Platelets < 50,000 mm^3^	4
Age ≥ 85 years *vs.* < 40 years	3.5
Liver failure (INR* > 1.5)	2.5
Severe renal failure (GFR** < 30 *vs.* ≥ 60 mL/min)	2.5
Admission to intensive care unit	2.5
Central venous catheter	2
Rheumatological disease	2
Active cancer	2
Age 40-84 *vs.* < 40 years	1.5
Male	1
Moderate renal failure (GFR** 30-59 *vs.* ≥60 mL/min)	1

* INR: International normalized ratio;

** GFR: glomerular filtration rate. Adapted from: Decousus et al.^6^

More than half of the episodes of major bleeding occurred in the 10% of hospitalized patients who had a bleeding risk score ≥ 7.^6^ The authors therefore defined an IMPROVE Bleeding Risk Score of ≥ 7 as high risk of bleeding and scores < 7 as low risk. Rates of major bleeding, compared with rates of any bleeding (defined as major or not major but clinically relevant) in patients with scores < 7 were 0.4% and 1.5% respectively. Among those with scores ≥ 7, the rate of major bleeding was 4.1% and the any bleeding rate was 7.9%.^6^

Mechanical prophylaxis was used more in patients with a bleeding score ≥ 7, than in patients with scores < 7 (16.3% *vs.* 8.9%, respectively). In contrast, pharmacological prophylaxis was used in similar proportions of patients with risk scores of < 7 and ≥ 7 (48.9% *vs.* 49.3%, respectively).^6^

This RAM therefore helps to make decisions on pharmacological or mechanical prophylaxis in medical patients at high risk of VTE.^6^ It can be used in combination with the IMPROVE score for VTE risk, enabling risk and benefit to be weighed up when choosing the best thromboprophylaxis strategy. This score has also been validated in other populations of medical patients.^35^^,^ 36

### Assessment of bleeding risk in surgical patients

Bleeding rates associated with pharmacological prophylaxis in surgical patients vary according to the profile of the surgery involved. A meta-analysis of 52 randomized studies of pharmacological VTE prophylaxis in general surgery patients reported that minor bleeding events are common, including hematoma at the administration site (~7%), wound hematoma (~6%), bleeding at drain sites (~2%), and hematuria (~2%).^37^ In contrast, major hemorrhagic complications were uncommon, including gastrointestinal (0.2%) or retroperitoneal bleeding (< 0.1%).^37^ Prophylaxis was withdrawn in 2% of patients and subsequent reoperation because of bleeding was needed in less than 1%. Notwithstanding, patients with one or more individual bleeding risk factors were considered high risk during the postoperative period.^37^

### Estimation of initial bleeding risk in surgical patients

The initial risk of bleeding has been poorly studied in non-orthopedic surgical patients. Major bleeding risk stratification was estimated according to the American College of Chest Physicians (ACCP) criteria in the following groups of surgical patients:^8^ general/abdominal/pelvic (~1%), bariatric (< 1%), plastic/reconstructive (0.5 to 1.8%), vascular (0.4 to 1.8%), cardiac (~5% [high risk]), thoracic (1%), neurosurgery/craniotomy (1 to 1.5%), spinal column (<0.5%), and severe trauma (3.4 to 4.7% [high risk]).

In orthopedic surgeries, estimates of initial bleeding risk in the absence of prophylaxis vary widely because of the heterogeneous characteristics of the populations involved and the surgical techniques employed.^38^ Risk of major bleeding is estimated in the range of 2 to 4% for orthopedic surgery with duration exceeding 45 minutes and in bilateral knee joint replacement. Non-major procedures, such as arthroscopies and shoulder, hand, and foot surgeries are considered lower bleeding risks (< 2%).^39^ Rates of major bleeding among patients given VTE prophylaxis varied from 0.1% to 3.1% in studies of hip joint replacement and from 0.2% to 1.4% in studies of knee joint replacement, suggesting that anticoagulants have little impact on risk of bleeding in these groups of patients.^40^

### Risk of bleeding in special situations

#### Thrombocytopenia

Current VTE prophylaxis guidelines are based on randomized clinical trials that exclude people who have a high risk of potential bleeding, thereby limiting specific recommendations on pharmacological prophylaxis for patients with thrombocytopenia and/or platelet dysfunction.^41^ These conditions are present in at least 25% of hospitalized individuals, represented by several pathologies, such as idiopathic thrombocytopenic purpura, thrombotic thrombocytopenic purpura, antiphospholipid antibody syndrome (APS), HIT, disseminated intravascular coagulation, drug-induced thrombocytopenia, liver, kidney, and bone marrow failure, and cancer.^41^ The minimum platelet levels recommended for pharmacological prophylaxis are also not uniform, ranging from 50,000 to 100,000/mm^3^.^6^^,^ 9 ^,^ 11 ^,^ 15 ^,^ 21 The IMPROVE Bleeding Risk Score^6^ defines 50,000/mm^3^ as the reference limit for platelets, whereas the NICE guidelines^21^ set the cutoff point at 75,000/mm^3^. The risk of spontaneous bleeding increases dramatically when platelet counts ranges from < 10,000 to 20,000/mm^3^, varying according to the cause of thrombocytopenia.^41^

#### Chronic liver disease

Thrombocytopenia or platelet dysfunction combined with coagulation abnormalities are common in patients with liver cirrhosis.^41^ However, these patients have a high incidence of portal and idiopathic venous thrombosis, showing that cirrhotic coagulopathy does not protect against thrombosis.^41^ Situations associated with mild to moderate thrombocytopenia (> 50,000/mm^3^) should not affect VTE prevention decisions. However, in patients with severe thrombocytopenia (< 50,000/mm^3^), prophylaxis should be considered on a case-by-case basis.^41^ Tufano et al.^41^ conducted a systematic review of thromboprophylaxis and thrombocytopenia, proposing specific recommendations for use of pharmacological prophylaxis (Table 3).

**Table 3:** Strategy for VTE prevention in patients with cirrhosis and/or thrombocytopenia.

**Risk of spontaneous bleeding**	**Recommendations**
Low (platelets < 90,000 mm^3^)	Pharmacological prophylaxis*
Intermediate (platelets from 50,000 to 90,000 mm^3^)	Pharmacological prophylaxis*
High (platelets < 50,000 mm^3^)	Pharmacological prophylaxis in selected cases *
	Mechanical prophylaxis preferable**

* VTE prophylaxis should be administered if the patient has one or more additional VTE risk factors;

** Graduated elastic compression stockings, intermittent pneumatic compression devices and venous foot pumps. Adapted from: Tufano et al.^41^

#### Antiphospholipid Antibody Syndrome (APS)

In patients with both APS and thrombocytopenia, the tendency to thrombosis generally far outweighs the risk of bleeding.^41^ In this population, VTE prophylaxis should be evaluated, especially for those considered high risk, such as, for example, patients positive for all three antiphospholipid antibodies: lupus anticoagulant, anticardiolipin, and anti ß2 glycoprotein I (triple-positive).^41^ The Global APS Score (GAPSS) is akidney, and bone marrow failure, and RAM that analyzes the antiphospholipid antibody profile and cardiovascular risk factors and could be useful for assessing risk of thrombotic events in patients with systemic lupus erythematosus, but it has not yet been validated.^42^

Up to 30% of patients with APS may have thrombocytopenia (< 100,000/ mm^3^), but bleeding is rare and is normally associated with catastrophic APS, immune thrombocytopenia, or patients who produce antibodies against prothrombin or other coagulation factors.^42^

#### Cancer patients

Cancer is an important independent risk factor for development of VTE.^43^ On the other hand, patients with cancer are also prone to bleeding, associated with complications of tumors, increased frequency of surgical procedures, and thrombocytopenia associated with systemic chemotherapy, making VTE prevention a major challenge in this population. Venous thromboembolism prophylaxis should be considered in hospitalized cancer patients even when they have thrombocytopenia, especially for those who have multiple VTE risk factors.^43^^,^ 44 Pharmacological prophylaxis is recommended at the standard dose for patients with platelet levels > 80,000/mm^3^.^43^^,^ 44 If platelet counts are below 80,000/mm,^3^ management should be decided individually.^43^^,^ 44 Careful monitoring of the undesirable effects of anticoagulant use *vs.* the risk of VTE is recommended.^43^^,^ 44 In cases in which pharmacological prophylaxis is contraindicated, use of mechanical prophylaxis should be optimized.

#### Chronic Renal Failure (CRF)

From the point of view of coagulation state, CRF is a paradoxical disease. Although it increases the risk of VTE because of endothelial injury/dysfunction, initial platelet hyperreactivity, increased fibrin formation, and reduced fibrinolytic system activity, it also increases the risk of major hemorrhage as renal function progressively deteriorates and platelet aggregation and adhesion reduce as a consequence.^45^ While the IMPROVE Bleeding Risk Score^6^ assesses CRF according to its severity (1 point for moderate CRF and 2.5 points for severe CRF), the VTE RAMs for medical patients (Brazilian VTE Prevention Guidelines,^9^ and the Padua,^11^ Geneva,^13^ and IMPROVE^12^ scores) and for surgical patients (Caprini^15^ and Rogers^16^ scores) do not include CRF as a risk factor for thrombosis. The fragile equilibrium between increased risk of VTE and risk of hemorrhage makes pharmacological prophylaxis of VTE a particular challenge, especially in those with advanced CRF (creatinine clearance of 15-29 mL/min) or end-stage kidney failure (creatinine clearance < 15 mL/min), for a variety of reasons including the fact that there is no specific RAM for this group of patients.^45^

With regard to pharmacological prophylaxis, current evidences are insufficient to conclude that the use of UFH at a dose of 5,000 UI three times / day increases the risk of major and minor hemorrhagic events in patients with creatinine clearance <30 ml / min compared to patients without severely impaired kidney function, as well as enoxaparin significantly increase the risk of major bleeding compared to UFH in this patient profile.^44^

### How to proceed with patients at increased risk of bleeding

In the case of hospitalized patients who have a high risk of VTE associated with a high risk of bleeding or have contraindications for the use of anticoagulants, mechanical methods of preventing VTE, such as intermittent pneumatic compression, graduated compression stockings and venous foot pump, are recommended.^6^ When mechanical prophylaxis options are used, the transition to a pharmacological agent should be considered as soon as the risk of bleeding becomes low or is reversed.

## CONCLUSIONS

Appropriate use of pharmacological prophylaxis should be aligned with minimization of bleeding risk so that patients classified as at high risk of development of VTE may obtain real clinical benefit from thromboprophylaxis.

Several VTE prevention guidelines provide guidance on the main factors involved in the risk of bleeding. However, to date, the only validated RAM that enables identification at hospital admission of medical patients at risk of bleeding is the IMPROVE Bleeding Risk Score.^6^^,^ 36 Patients with scores < 7 can safely be given pharmacological prophylaxis.^6^ In contrast, prophylaxis decisions on patients at high risk of bleeding (scores ≥ 7) who also simultaneously have a high risk of VTE should be taken individually and dynamically over the course of the hospital stay, up to hospital discharge. In patients undergoing surgery, it is necessary to consider the procedure’s potential risk of bleeding in conjunction with individual risk factors to define the best VTE prevention strategy.
