# Supplementary material for: Bleeding risk assessment for venous thromboembolism prophylaxis
Source: J Vasc Bras. 2021 Apr 28;20:e20200109. doi: 10.1590/1677-5449.200109 (PMC8147884; doi:10.1590/1677-5449.200109)
Supplement: Translation

# Translation: Avaliação do risco de sangramento na profilaxia do tromboembolismo venoso

**Authors:** Maria Chiara Chindamo, Marcos Arêas Marques

**Affiliations:** 1 Universidade Federal do Rio de Janeiro – UFRJ, Rio de Janeiro, RJ, Brasil.; 2 Hospital Barra D'Or - Rede D'Or São Luiz , Rio de Janeiro, RJ, Brasil.; 3 Universidade do Estado do Rio de Janeiro – UERJ, RJ, Brasil.

**Keywords:** trombose venosa, embolia pulmonar, profilaxia, hemorragia, segurança do paciente, avaliação de risco

## Abstract

O tromboembolismo venoso (TEV) é uma das principais causas preveníveis de morbimortalidade em pacientes hospitalizados, sendo a embolia pulmonar (EP) fatal possivelmente a sua primeira manifestação. Diretrizes nacionais e internacionais recomendam o uso de modelos de avaliação de risco para a prescrição de profilaxia do TEV em pacientes hospitalizados. Apesar das evidências e diretrizes de apoio, o uso da tromboprofilaxia permanece abaixo do ideal, o que pode resultar da baixa conscientização dos benefícios da profilaxia, mas também pode refletir o medo de complicações hemorrágicas, justificando a subutilização da tromboprofilaxia em todo o mundo. A avaliação do risco de sangramento é, portanto, necessária para a adequação de profilaxia e deve ser realizada de forma concomitante à avaliação do risco de trombose. O objetivo desta revisão é salientar a importância da avaliação conjunta do risco de TEV e do risco de sangramento em pacientes hospitalizados.

## INTRODUÇÃO 

O tromboembolismo venoso (TEV) é uma das principais causas preveníveis de morbimortalidade em pacientes hospitalizados^1^^,^ 2. A embolia pulmonar (EP) fatal é frequentemente a primeira manifestação de TEV e pode representar até 10% de todas as mortes hospitalares^2^^,^ 3. Os pacientes hospitalizados apresentam risco de TEV devido à presença de fatores adquiridos ou hereditários, como obesidade, câncer, TEV prévio, trombofilias, trauma, cirurgia, infarto agudo do miocárdio, acidente vascular cerebral, idade avançada, insuficiência cardíaca congestiva, infecção aguda, imobilidade e admissão em unidades de terapia intensiva, entre outros^4^^-^ 6.

Diretrizes nacionais e internacionais recomendam o uso de modelos de avaliação de risco [*risk assessment model* (MAR)] para a profilaxia farmacológica ou mecânica em pacientes clínicos^7^^-^ 14, cirúrgicos^8^^,^ 15 ^,^ 16 ou obstétricos^10^, visando à melhor estratégia de prevenção. Contudo, o risco de TEV não pode ser avaliado isoladamente. O risco de sangramento, induzido ou potencializado pelos anticoagulantes, também deve ser avaliado simultaneamente quando se considera a adequação da tromboprofilaxia^6^^,^ 7 ^,^ 9.

Embora diversos estudos reportem baixas taxas de sangramento relacionadas à farmacoprofilaxia^11^^,^ 17 ^,^ 18, o medo de eventos hemorrágicos é uma das principais justificativas da sua subutilização em todo o mundo^6^. A identificação de condições com potencial risco de sangramento e a implementação de MAR são, portanto, fundamentais para o uso adequado da tromboprofilaxia^19^.

O objetivo desta revisão é salientar a importância da avaliação conjunta do risco de TEV e do risco de sangramento em pacientes hospitalizados.

## AVALIAÇÃO DE RISCO TROMBÓTICO *VERSUS* RISCO DE SANGRAMENTO

Inúmeros MARs de TEV estão disponíveis, tanto para pacientes clínicos quanto cirúrgicos, e direcionam as principais recomendações de tromboprofilaxia com base na estratificação de risco^7^^-^ 16. O modelo mais adequado de avaliação ainda não está definido^19^. Na estratificação de risco de TEV, devemos utilizar um modelo validado para a população em questão aplicado de forma sistemática nas principais etapas de assistência: admissão hospitalar, transição entre setores e alta hospitalar. Essa última avaliação é particularmente importante nos pacientes que mantêm fatores de risco para TEV na alta, como, por exemplo, a imobilidade prolongada^2^. A escolha da melhor estratégia de tromboprofilaxia deve considerar, simultaneamente, o risco de TEV e o risco potencial de sangramento^20^^,^ 21.

São consideradas contraindicações absolutas ao uso de anticoagulantes o sangramento ativo, grave ou potencialmente fatal, não reversível com intervenção médica ou cirúrgica, incluindo qualquer sangramento ativo em local crítico (intracraniano, pericárdico, retroperitoneal, intraocular, intra-articular e intraespinhal), hipertensão arterial maligna não controlada, coagulopatia grave não compensada, disfunção plaquetária ou distúrbios primários de hemostasia graves, trombocitopenia persistente (< 20.000/mm^3^) e procedimentos invasivos de alto risco em local crítico, como punção lombar e raquianestesia em pacientes cujo procedimento cirúrgico é planejado no período de 6 a 12 horas seguintes^20^. Outros fatores associados ao risco aumentado de sangramento são a trombocitopenia induzida pela heparina (TIH), o uso concomitante de antiagregantes plaquetários e/ou anti-inflamatórios não hormonais e a disfunção renal, principalmente quando utilizados anticoagulantes de eliminação renal [heparina de baixo peso molecular (HBPM) e fondaparinux]^15^. Em pacientes que apresentam *clearance* de creatinina < 30 mL/min, recomenda-se a redução da dose de HBPM, monitoramento da ação anticoagulante ou substituição por heparina não fracionada (HNF)^4^. A revisão periódica de ambos os riscos, principalmente nas mudanças de quadro clínico, ajuda a orientar a melhor estratégia para a profilaxia^21^ (Figura 1).

**Figura 1:** Recomendações de profilaxia de tromboembolismo venoso (TEV) segundo estratificação do risco de TEV *versus* risco de sangramento. Adaptado de: National Institute for Health and Care Excellence – NICE. NG89^21^.

### Modelos de avaliação de risco de TEV

Entre os principais MARs de TEV em pacientes clínicos, estão a Diretriz Brasileira de Prevenção de TEV em pacientes clínicos hospitalizados^9^ e os escores de Padua^11^, Genebra^13^ e IMPROVE (*International Medical Prevention Registry on Venous Thromboembolism*)^12^. Para a avaliação de pacientes cirúrgicos, são recomendados os escores de Caprini^15^ e Rogers^16^, que definem o risco de TEV com base nas características dos pacientes e no perfil de cada cirurgia. Mulheres hospitalizadas durante a gravidez, no puerpério ou no período de até seis semanas após aborto espontâneo ou interrupção da gravidez, devem ser avaliadas quanto à necessidade de farmacoprofilaxia^21^. O MAR mais utilizado nesse perfil de pacientes foi desenvolvido pelo *Royal College of Obstetricians and Gynecologists* (RCOG)^10^^,^ 21.

Apesar da recomendação de avaliação concomitante do risco de TEV *versus* risco de sangramento durante o cuidado do paciente hospitalizado, são escassos os MARs de sangramento no contexto da profilaxia de TEV^19^. Poucos são os modelos que associam essas duas características^9^^,^ 12 ^,^ 21 (Tabela 1).

**Tabela 1:** Lista de modelos de avaliação de risco (MAR) de tromboembolismo venoso (TEV) e de sangramento de acordo com a população estudada^9^^-^ 16 ^,^ 21.

**MAR**	**Tipo de paciente**	**Risco de sangramento**	**Recomendação de profilaxia**
Caprini, 2005	Cirúrgico	-	X
Geneva, 2006	Clínico	-	X
Rogers, 2007	Cirúrgico	-	X
Diretriz brasileira, 2007	Clínico	X	X
Padua, 2010	Clínico	-	X
IMPROVE, 2011	Clínico	X	X
UK RCOG, 2015	Obstétrico	-	X
NICE NG89, 2018	Clínico/cirúrgico	X	X

Esse cenário é bastante distinto daquele encontrado na avaliação de risco de sangramento aplicada à anticoagulação plena na prevenção dos fenômenos tromboembólicos da fibrilação atrial ou no tratamento de TEV. Dez MARs de sangramento estão disponíveis com esse objetivo. Seis deles são aplicáveis aos casos dos pacientes que fazem uso de anticoagulantes orais na fibrilação atrial (ABC^22^, ORBIT^23^, ATRIA^24^, HAS-BLED^25^, HEMORR_2_HAGES^26^, Shireman^27^), três no tratamento anticoagulante de TEV (VTE-BLED^28^, Ruiz-Gimenez^29^, Kuijer^30^) e um misto (OBRI^31^). Esses escores evidenciam situações de aumento do risco de sangramento associado à anticoagulação plena e auxiliam na aplicação de estratégias que ajudam a minimizar o risco de hemorragia por meio da intervenção em fatores de risco modificáveis^32^.

## RISCO DE SANGRAMENTO NA FARMACOPROFILAXIA

### Avaliação de risco de sangramento em pacientes clínicos

#### IMPROVE *Bleeding Risk Score*

O principal MAR de sangramento associado à farmacoprofilaxia em pacientes clínicos hospitalizados é o IMPROVE *Bleeding Risk Score* 6 ^,^ 7. Decousus et al.^6^ utilizaram uma análise multivariada para identificar e pontuar fatores na admissão hospitalar associados ao risco de sangramento em pacientes clínicos agudamente doentes. Com base nos dados do IMPROVE^12^, um estudo observacional multicêntrico desenvolvido para avaliar os padrões de profilaxia de TEV em mais de 15.000 pacientes clínicos, os autores verificaram a incidência de sangramento e identificaram os fatores, na admissão, associados ao risco de sangramento^6^. O sangramento maior foi definido como sangramento fatal e/ou sintomático em uma área ou órgão crítico e/ou sangramento que resultou em queda de hemoglobina ≥ 2 g/dL ou levou à transfusão de dois ou mais concentrados de hemácias^6^. O sangramento não maior, mas clinicamente relevante, foi caracterizado como hemorragia gastrointestinal, hematúria macroscópica de duração > 24 h, epistaxe substancial com necessidade de intervenção, epistaxe recorrente e/ou de duração de pelo menos cinco minutos, hematomas extensos (> 5 cm de diâmetro), sangramento intra-articular, menorragia ou metrorragia ou ainda outro sangramento importante que necessitasse de intervenção médica^6^^,^ 33.

A incidência cumulativa de sangramentos hospitalares descritos como maiores e não maiores até 14 dias após a internação foi de 3,2% (1,2% de sangramento maior e 2,0% de sangramento não maior clinicamente relevante)^6^.

Os fatores de risco, na admissão, independentemente associados ao risco de sangramento foram^6^: úlcera gastroduodenal ativa, sangramento nos três meses anteriores à admissão e contagem de plaquetas < 50.000/mm^3^. Outros fatores de risco de sangramento incluíram idade avançada, insuficiência hepática e/ou renal, permanência em unidade de terapia intensiva, presença de cateter venoso central, doença reumática, câncer e sexo masculino, fatores que também estão relacionados ao aumento do risco de TEV^6^. Cada um dos fatores acima, com ponderação apropriada, foi inserido neste MAR (Tabela 2). Os autores também elaboraram um recurso *on-line* no qual o risco de sangramento pode ser avaliado^34^.

**Tabela 2:** IMPROVE *Bleeding Risk Score*.

**Fatores de risco**	**Pontuação**
Úlcera gastroduodenal ativa	4,5
Hemorragia três meses antes da internação	4
Plaquetas < 50.000 mm^3^	4
Idade ≥ 85 anos *versus.* < 40 anos	3,5
Insuficiência hepática (RNI* > 1,5)	2,5
Insuficiência renal severa (TFG** < 30 *versus.* ≥ 60 mL/min)	2,5
Internação em unidade de terapia intensiva	2,5
Cateter venoso central	2
Doença reumatológica	2
Câncer ativo	2
Idade 40-84 *versus* < 40 anos	1,5
Sexo masculino	1
Insuficiência renal moderada (TFG** 30-59 *versus* ≥60 mL/min)	1

* RNI: Razão Normalizada Internacional;

** TFG: taxa de filtração glomerular. Adaptada de: Decousus et al.^6^.

Mais da metade dos episódios de sangramento maior ocorreu em 10% dos pacientes hospitalizados que tiveram escore de risco de sangramento ≥ 7^6^. Desse modo, os autores consideraram como alto risco de sangramento a pontuação no IMPROVE *Bleeding Risk Score* ≥ 7 e como baixo risco a pontuação < 7. As taxas de sangramento maior, em comparação às taxas de qualquer sangramento (definido como maior ou não maior clinicamente relevante) nos pacientes com escore < 7 foram respectivamente 0,4% e 1,5%. Naqueles com escore ≥ 7, a taxa de sangramento maior foi de 4,1% e a de qualquer sangramento, 7,9%^6^.

Profilaxia mecânica foi mais utilizada em pacientes com escore de sangramento ≥ 7, comparada ao escore < 7 (16,3% *versus* 8,9%, respectivamente). Por outro lado, a profilaxia farmacológica foi utilizada em proporções semelhantes em pacientes com escore de risco de < 7 e ≥ 7 (48,9% *versus* 49,3%, respectivamente)^6^.

Este MAR ajuda, portanto, a decidir sobre a profilaxia farmacológica ou mecânica em pacientes clínicos de alto risco de TEV^6^. Pode ser aplicado em associação com o escore IMPROVE para risco de TEV, permitindo a ponderação do risco e do benefício na escolha da melhor estratégia de tromboprofilaxia. Esse escore já foi validado em outras populações de pacientes clínicos^35^^,^ 36.

### Avaliação de risco de sangramento em pacientes cirúrgicos

A taxa de sangramento associada à farmacoprofilaxia em pacientes cirúrgicos varia de acordo com o perfil da cirurgia. Uma metanálise de 52 estudos randomizados sobre farmacoprofilaxia de TEV em pacientes de cirurgia geral relatou que os sangramentos menores são comuns e abrangem hematomas no local de aplicação (~7%), hematoma da ferida (~6%), sangramento no local do dreno (~2%) e hematúria (~2%)^37^. As complicações hemorrágicas maiores, no entanto, foram incomuns e incluíram sangramento gastrointestinal (0,2%) ou retroperitoneal (< 0,1%)^37^. A descontinuação da profilaxia ocorreu em 2% dos pacientes e a reoperação subsequente por sangramento em menos de 1%. Da mesma forma, os pacientes com um ou mais fatores de risco individuais de sangramento foram considerados de alto risco no pós-operatório^37^.

### Estimativas do risco inicial de sangramento em cirurgia

O risco inicial de sangramento foi pouco estudado em pacientes cirúrgicos não ortopédicos. A estratificação de risco de sangramento maior foi estimada pelo *American College of Chest Physicians* (ACCP) nos seguintes grupos de pacientes cirúrgicos^8^: geral/abdominal/pélvica (~1%), bariátrica (< 1%), plástica/reconstrutiva (0,5 a 1,8%), vascular (0,4 a 1,8%), cardíaca [~5% (alto risco)], torácica (1%), neurocirurgia/craniotomia (1 a 1,5%), coluna vertebral (<0,5%) e trauma grave [3,4 a 4,7% (alto risco)].

Nas cirurgias ortopédicas, as estimativas de risco inicial de sangramento na ausência de profilaxia são heterogêneas devido às características distintas das populações e das técnicas cirúrgicas utilizadas^38^. Estima-se um risco de sangramento maior, variando de 2 a 4% nas cirurgias ortopédicas com duração acima de 45 minutos e na artroplastia bilateral de joelhos. Procedimentos menores, como artroscopias, cirurgias de ombro, mãos e pés, são considerados de menor risco de sangramento (< 2%)^39^. As taxas de sangramento maior em pacientes submetidos a profilaxia de TEV variaram de 0,1% a 3,1% nos estudos de artroplastia de quadril e de 0,2% a 1,4% nos estudos de artroplastia de joelho, sugerindo haver pouca interferência dos anticoagulantes no risco de sangramento desses grupos de pacientes^40^.

### Risco de sangramento em situações especiais

#### Trombocitopenia

As diretrizes atuais sobre profilaxia de TEV são baseadas em ensaios clínicos randomizados que excluem os indivíduos de risco potencialmente alto de sangramento, limitando, portanto, as recomendações específicas sobre farmacoprofilaxia em pacientes com trombocitopenia e/ou disfunção plaquetária^41^. Essas condições estão presentes em pelo menos 25% dos indivíduos hospitalizados e são representadas por uma série de patologias, como púrpura trombocitopênica idiopática, púrpura trombocitopênica trombótica, síndrome do anticorpo antifosfolipídeo (SAF), TIH, coagulação intravascular disseminada, plaquetopenia induzida por medicamentos, insuficiência hepática, renal e medular e câncer^41^. Os valores mínimos de plaquetas para a utilização da farmacoprofilaxia também não são uniformes, variando entre 50.000 e 100.000/mm^3^.^6^^,^ 9 ^,^ 11 ^,^ 15 ^,^ 21. O IMPROVE *Bleeding Risk Score* 6 considera como referência o limite de plaquetas de 50.000/mm^3^, enquanto a diretriz NICE^21^ estabelece o ponto de corte em 75.000/mm^3^. O risco de sangramento espontâneo aumenta dramaticamente quando há contagens de plaquetas de < 10.000 a 20.000/mm^3^ e varia de acordo com a causa da trombocitopenia^41^.

#### Hepatopatia crônica

A trombocitopenia ou disfunção plaquetária associadas a anormalidades da coagulação são comuns em pacientes com cirrose hepática^41^. No entanto, esses pacientes apresentam alta incidência de trombose venosa portal e idiopática, o que significa que a coagulopatia da cirrose não protege contra a trombose^41^. As situações associadas à trombocitopenia leve/moderada (> 50.000/mm^3^) não devem interferir nas decisões de prevenção de TEV. Na trombocitopenia grave (< 50.000/mm^3^), no entanto, a profilaxia deve ser considerada individualmente^41^. Em uma revisão sistemática sobre tromboprofilaxia e trombocitopenia, Tufano et al.^41^ propõem recomendações especificas para o uso de profilaxia farmacológica (Tabela 3).

**Tabela 3:** Estratégia de prevenção do TEV em pacientes com cirrose e/ou trombocitopenia.

**Risco de sangramento espontâneo**	**Recomendações**
Baixo (plaquetas < 90.000 mm^3^)	Profilaxia farmacológica*
Intermediário (plaquetas ente 50 e 90.000 mm^3^)	Profilaxia farmacológica*
Alto (plaquetas < 50.000 mm^3^)	Profilaxia farmacológica em casos selecionados*
	Profilaxia mecânica preferencial**

* A profilaxia do TEV deve ser realizada na presença de um ou mais fatores de risco adicionais para TEV;

** Meias elásticas de compressão graduada, compressão pneumática intermitente e dispositivos e bombas podais. Adaptada de: Tufano et al.^41^.

#### Síndrome anticorpo antifosfolipídeo (SAF)

Em pacientes com SAF e trombocitopenia, a tendência trombótica é geralmente muito superior ao risco de sangramento^41^. A profilaxia de TEV deve ser avaliada nessa população, especialmente naqueles considerados de alto risco, como, por exemplo, os pacientes positivos para os três anticorpos antifosfolipídios: anticoagulante lúpico, anticardiolipina e anti ß2 glicoproteína I (triplo-positivo)^41^. O *Global APS Score* (GAPSS) é um MAR que analisa o perfil dos anticorpos antifosfolipídios e fatores de risco cardiovascular e pode ser útil para avaliar o risco de eventos trombóticos em pacientes portadores de lúpus eritematoso sistêmico, porém ainda não foi validado^42^.

Até 30% dos pacientes portadores de SAF podem apresentar trombocitopenia (< 100.000/ mm^3^), porém o sangramento é raro e normalmente está associado à SAF catastrófica, trombocitopenia imune ou naqueles que desenvolvem anticorpos contra protrombina ou outros fatores da coagulação^42^.

#### Pacientes oncológicos

O câncer é um fator de risco independente e importante para o desenvolvimento de TEV^43^. Por outro lado, pacientes com câncer são propensos a sangramentos, associados às complicações dos tumores, frequência aumentada de procedimentos cirúrgicos e trombocitopenia associada à quimioterapia sistêmica, tornando a prevenção do TEV um grande desafio nessa população. A profilaxia do TEV deve ser considerada em pacientes hospitalizados com câncer mesmo na presença de trombocitopenia, principalmente nos que têm múltiplos fatores de risco para TEV^43^^,^ 44. Nos pacientes que apresentam valores de plaquetas > 80.000/mm^3^, recomenda-se o uso de farmacoprofilaxia em dose padrão^43^^,^ 44. Se a contagem de plaquetas estiver abaixo de 80.000/mm^3^, a conduta deve ser considerada individualmente^43^^,^ 44. Recomenda-se monitoramento cuidadoso quanto a efeitos indesejáveis relacionados ao uso do anticoagulante *versus* risco de TEV^43^^,^ 44. No caso de contraindicação à profilaxia farmacológica, deve-se otimizar o uso da profilaxia mecânica.

#### Insuficiência Renal Crônica (IRC)

A IRC é uma doença de caráter paradoxal do ponto de vista da coagulação, pois, ao mesmo tempo em que aumenta o risco de TEV devido à injúria/disfunção endotelial, hiperreatividade plaquetária inicial, aumento da formação de fibrina e diminuição da atividade do sistema fibrinolítico, aumenta o risco de hemorragia maior com a piora progressiva da função renal e consequente decréscimo da adesão e agregação plaquetária^45^. Embora o IMPROVE *Bleeding Risk Score* 6 pontue a IRC de acordo com a sua gravidade (um ponto para IRC moderada e 2,5 pontos para IRC severa), os MARs de TEV para pacientes clínicos (Diretriz Brasileira de Prevenção-de TEV^9^ e os escores de Padua^11^, Genebra^13^ e IMPROVE^12^) e pacientes cirúrgicos (escore de Caprini^15^ e Rogers^16^) não incluem a IRC como fator de risco de trombose. Esse equilíbrio frágil entre o risco aumentado de TEV e o risco de hemorragia torna a farmacoprofilaxia do TEV um desafio particular especialmente naqueles com IRC avançada (*clearance* de creatinina 15-29 mL/min) ou falência renal terminal (clearance de creatinina < 15 mL/min), por várias razões, incluindo o fato de não haver um MAR específico para esse grupo de pacientes^45^.

Com relaçao às opções de profilaxia farmacológica, as evidências são insuficientes para concluir que o uso de HNF na dose de 5.000 UI três vezes/dia aumente o risco de eventos hemorrágicos graves e menores em pacientes com *clearence* de creatinina < 30 mL/min em comparação a pacientes sem disfunção renal severa ou que a enoxaparina aumente significativamente o risco de sangramento maior em comparação a HNF nesse perfil de pacientes^46^.

### Como proceder no caso de pacientes com aumento do risco de sangramento

No caso dos pacientes hospitalizados que apresentam alto risco de TEV associado a alto risco de sangramento ou que têm contraindicação do uso de anticoagulantes, os métodos mecânicos de prevenção de TEV, como compressão pneumática intermitente, meias de compressão graduadas e bomba venosa plantar são recomendados^6^. Quando as opções de profilaxia mecânica são utilizadas, a transição para um agente farmacológico deve ser considerada assim que o risco de sangramento se tornar baixo ou for revertido.

## CONCLUSÃO

A adequação do uso de farmacoprofilaxia deve estar alinhada com a minimização do risco de sangramento para que os pacientes classificados como de alto risco de desenvolvimento de TEV obtenham benefício clínico real da tromboprofilaxia.

Diversas diretrizes de prevenção de TEV orientam sobre os principais fatores implicados no risco de sangramento. No entanto, até o momento, o único MAR validado que permite a identificação dos pacientes clínicos na admissão hospitalar sob risco de sangramento é o IMPROVE *Bleeding Risk Score* 6 ^,^ 36. Os pacientes com pontuação < 7 podem receber farmacoprofilaxia com segurança^6^. Por outro lado, pacientes com alto risco de sangramento (pontuação ≥ 7), mas que apresentam simultaneamente alto risco de TEV, devem ter as decisões relativas à profilaxia avaliadas de forma individualizada e dinâmica ao longo da internação, até a alta hospitalar. Nos pacientes submetidos à cirurgia, é necessário considerar o potencial risco de sangramento do procedimento em conjunto com os fatores de risco individuais para definir a melhor estratégia de prevenção de TEV.
